# A Cellulose-Type Carrier for Intimate Coupling Photocatalysis and Biodegradation

**DOI:** 10.3390/polym14152998

**Published:** 2022-07-24

**Authors:** Zhou Wan, Chunlin Jiao, Qilin Feng, Jue Wang, Jianhua Xiong, Guoning Chen, Shuangfei Wang, Hongxiang Zhu

**Affiliations:** 1 School of Resources, Environment and Materials, Guangxi University, Nanning 530004, China; wulizhouzhou@icloud.com (Z.W.); m13171793629@163.com (C.J.); rosalin28@126.com (Q.F.); wangjuecynthia@163.com (J.W.); 2 Guangxi Bossco Environmental Protection Technology Co., Ltd., Nanning 530007, China; 3 Guangxi Key Laboratory of Clean Pulp & Papermaking and Pollution Control, Nanning 530004, China; wangsf@gxu.edu.cn (S.W.); zhx@gxu.edu.cn (H.Z.)

**Keywords:** carrier, cellulose, degradation, photocatalysis, 1,2,4-trichlorobenzene

## Abstract

Intimate coupling photocatalysis and biodegradation treatment technology is an emerging technology in the treatment of refractory organic matter, and the carrier plays an important role in this technology. In this paper, sugarcane cellulose was used as the basic skeleton, absorbent cotton was used as a reinforcing agent, anhydrous sodium sulfate was used as a pore-forming agent to prepare a cellulose porous support with good photocatalytic performance, and nano-TiO_2_ was loaded onto it by a low-temperature bonding method. The results showed that the optimal preparation conditions of cellulose carriers were: cellulose mass fraction 1.0%; absorbent cotton 0.6 g; and Na_2_SO_4_ 60 g. The SEM, EDS and XPS characterization further indicated that the nano-TiO_2_ was uniformly loaded onto the cellulose support. The degradation experiments of Rhodamine B showed that the nano-TiO_2_-loaded composite supports had good photocatalytic performance. The degradation rate of 1,2,4-trichlorobenzene was more than 92% after 6 cycles, and the experiment of adhering a large number of microorganisms on the carriers before and after the reaction showed that the cellulose-based carriers obtained the required photocatalytic performance and stability, which is a good cellulose porous carrier.

## 1. Introduction

Intimate coupling photocatalysis and biodegradation (ICPB) technology [1,2] is an emerging processing technology that successfully combines photocatalytic technology and biological processing technology. In the meantime, it also integrates the advantages of both advanced oxidation technology and biodegradation technology [3], which has synergistic effects [4], and it has a good effect on the treatment of difficult-to-degrade pollutants.

**Figure 1:**
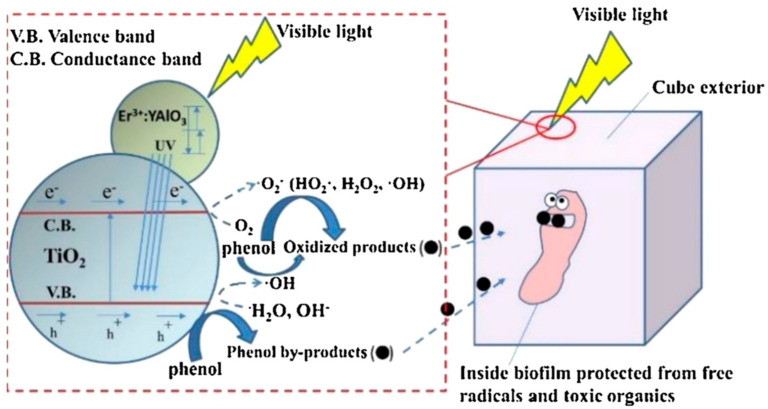
Schematic of the principle of pollutants degradation in ICPB.

The principle of pollutants degradation in ICPB is shown in Figure 1 [5]. Such active species with strong oxidizability as hydroxyl radical, superoxide radicals and holes [6,7,8], generated from a catalyst on the surface of carriers under light, decompose pollutants into simple and easy-biodegradable intermediate products. These products will transform into carbon dioxide and water by microbial metabolism in carriers, not excluding the direct degradation of pollutants. When considering studies related to tetracycline [9], 4-chlorophenol [10,11], methylene [12] and other pollutants in the ICPB system, a synergistic effect can be found among adsorption, photocatalysis and biodegradation [13]. The adsorption of pollutants by carriers enables the active species to oxidize and decompose pollutants in time, which reduces the damage to microorganisms from active species. The mineralization of intermediate products by microorganisms, in return, alleviates the competitive consumption of active species and improves the photocatalytic efficiency. Consequently, the porous carriers are crucial to the successful construction and operation of ICPB system.

The porous carriers involved in ICPB system mainly contain porous ceramic [14,15], cellulose [2] and the polyurethane sponge carriers [16]. Ceramic carriers have the advantage of strong stability, reusability and durability, etc. Despite this, it is difficult to ensure that carriers flow in a reactor, which makes the operational mode of the cycle between photocatalysis and microbial degradation [17]. Except for the good adsorption performance and stability [18], the lower density of polyurethane sponge-type carriers is conducive to running with the current. However, it is difficult to maintain biofilm stabilization because the hydraulic sheared and recycling process is complicated. The significant adsorption performance of cellulose carriers is favorable for attaching the catalyst and microorganism, while the cellulose material is bio-friendly and will not cause secondary pollution. However, the original structure of carriers may be damaged by microorganism degradation for a long-term operation.

Considering that the method of ICPB has great potential and research value for the degradation of persistent organic pollutants, this paper selects bagasse cellulose and absorbent cotton as major materials to prepare porous carriers; it also constructs a further ICPB system to explore the possibility of 1,2,4-trichlorobenzene (1,2,4-TCB) degradation in ICPB systems and to perfect a theoretical basis and possible practical methods for degradation of persistent organic pollutants. As a typical AOX pollutant, 1,2,4-TCB is widely present in bleaching wastewater, herbicides and other pesticide wastewater, and with stable physical and chemical properties it can exist stably in water and soil environments for a long time [19]; it is also toxic to animals, plants and humans [20]. Therefore, it is particularly important to carry out research on the degradation of 1,2,4-TCB.

## 2. Materials and Methods

### 2.1. Materials

The materials obtained were: sugarcane cellulose from Guangxi Guigang Guitang Co., Ltd.; visible light-responsive titanium dioxide (nano-TiO_2_) from Liuzhou Rose Nanomaterials Technology Co., Ltd.; zinc chloride from Tianjin Ou Boke Chemical Sales Co., Ltd.; sodium sulfate from Guangdong Guanghua Sci-Tech Co, Ltd.; absorbent cotton from Nanchang Leiyi Medical Appliance Co., Ltd.; Rhodamine B (RhB) from Aladdin Reagent Co., Ltd. (Shanghai, China); and 1,2,4-TCB from Macklin. All chemicals were analytically pure. Ammonium chloride (NH_4_Cl), disodium hydrogen phosphate (Na_2_HPO_4_·12H_2_O), sodium dihydrogen phosphate (NaH_2_PO_4_·2H_2_O) and magnesium sulfate (MgSO_4_·7H_2_O) were purchased from Guangdong Chemical Reagent Engineering Technology Research and Development Center; and calcium chloride (CaCl_2_) and ferric chloride (FeCl_2_·6H_2_O) were purchased from the Sinopharm Group. Activated sludge came from the research center of the Guangxi Bossco Environmental Protection Technology Co., Ltd.

### 2.2. Preparation of Cellulose-Type Carriers

The cellulose carriers was prepared for bagasse cellulose, absorbent cotton and sodium sulfate (Na_2_SO_4_) in zinc chloride (ZnCl_2_) solution. After solidifying in deionized water and being freeze-dried, the prepared carrier had a large number of pores. The specific process was as followed: (1) mixture (100 g) stirred for 60 min at a temperature of 80 ℃, which included ZnCl_2_ (70%, wt) solution and cellulose with different mass rates in the mixture of 1%, 2%, 3%, 4%; (2) different dosages of absorbent cotton at 0.4 g, 0.5 g, 0.6 g, 0.7 g and 0.8 g were added into the mixture; (3) after stirring the mixture for 60 min at a temperature of 60 ℃, 40 g, 50 g, 60 g, 70 g and 80 g of Na_2_SO_4_ were added, respectively; after stirring for 60 min at a temperature of 60 ℃, the mixture was solidified in deionized water for 2 days and freeze-dried for 2 days at a temperature of −70 ℃. The carriers with a size of 5 mm×5 mm×5 mms were then obtained.

Using selected water absorption, wet density, porosity and retention rates as indicators of performance, the optimal conditions for the prepared carriers were analyzed. The calculation method was as followed: absorb surface moisture by filter papers after soaking the prepared carriers in deionized water for 24 h and weighing its wet weight (*m*_1_); measured total volume (*V*_1_) of carriers by the drainage in cylinder (100 mL, with accuracy of 1 mL); weigh the dry weight (*m*_0_) of the carriers after drying for 6 h at a temperature of 60 ℃ in a vacuum drying oven; stir the mixture of water and carriers for 60 min at a speed of 500 r/min in a beaker (1 L, with 600 mL water), in which carriers were added by the volume ratio of 1/15 (carrier/water); after stirring, measure the total volume (*V*_2_) of the carriers again. The wet density (*ρ*, g/cm^3^), water absorption (*ω*, %), porosity (*ε*, %) and retention rates (*σ*, %) were then calculated using the following Equation [21]:

(1) $$\;\rho =\frac{m_{1}}{V_{0}}$$

(2) $$\omega =\frac{m_{1}-m_{0}}{m_{0}}\times 100\%$$

(3) $$\varepsilon =\frac{m_{1}-m_{0}}{\rho_{Aq}V_{1}}\times 100\%$$

(4) $$\sigma =\frac{V_{2}}{V_{1}}\times 100\%$$

### 2.3. Photocatalytic Performance of Cellulose Support

A xenon lamp (XHA250W, Spectrum 200 nm–1100 nm) was used as the light source; the 15 mg/L RhB solution was placed under the lamp for 5 h, and the TiO_2_-loaded carriers were added to carry out the photocatalytic degradation of RhB to detect the TiO_2_ loading. Experiments of 4 cycles of degradation of RhB solution were carried out to test the reusability of the carriers.

### 2.4. System Construction of ICPB

The catalyst was loaded onto carriers via a simple and efficient low-temperature process on the basis of previous research [22]: dissolve 1.5 g visible light-responsive titanium dioxide (nano-TiO_2_) in 15 mL solution of 0.3 g/L defused sodium and stir the mixture for 15 min; after soaking in the mixture in the first step for 10 min, bake the carriers for 120 min at a temperature of 60 ℃; ultrasonically clean the nano-TiO_2_-carriers in deionized water for 5 min, and repeat the process 3 times.

Activated sludge was used as the biological source and cultivated in a 2.0 L reactor. The hydraulic retention time was 24 h while the aeration rate was 0.8 L/min and the pH was 6–8. Domestication was finished after 31 days and increased by one gradient every three days with the concentration of 1,2,4-TCB from 0 mg/L to 18 mg/L. The prepared carriers were added to the activated sludge for microorganisms to attach [23]. Medium composition was as follows: NH_4_Cl (35.80 mg/L); Na_2_HPO_4_·12H_2_O (10.17 mg/L); NaH_2_PO_4_·2H_2_O (5.03 mg/L); MgSO_4_·7H_2_O (2.00 mg/L); CaCl_2_ (2.00 mg/L); FeCl_2_·6H_2_O (1.00 mg/L).

**Figure 2:**
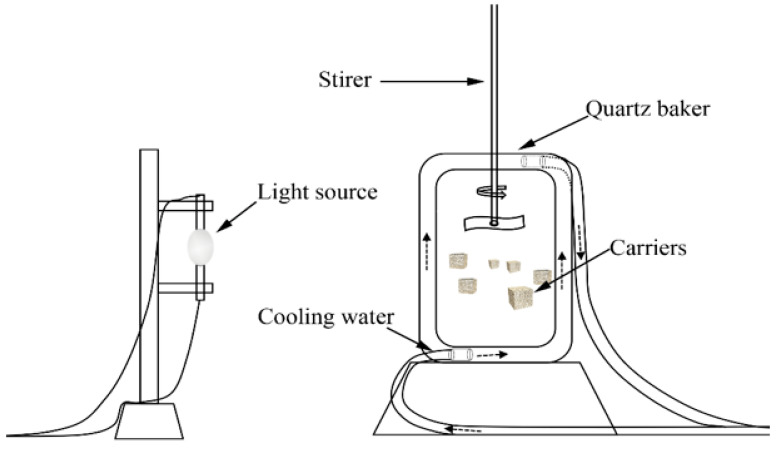
Schematic of the experimental setup.

The schematic diagram for the system of ICPB is shown in Figure 2. Using a xenon lamp (XHA250W) as a light source, place a quartz beaker (500 mL) containing 1,2,4-TCB solution at 15 cm of the xenon lamp, 300 mL of 1,2,4-TCB solution, and an initial concentration of 8.0 mg/ L. The carrier dosage (volume ratio) is 8%, the pH is 5, the stirring speed is 100 r/min, and the reaction time is 7 h.

### 2.5. Characterization

Scanning Electron Microscopy (SEM, Hitachi, Tokyo, Japan) and Energy Dispersive Spectrometry (EDS, Phenom, ThermoFisher, Waltham, MA, USA) were used to investigate morphology and surface properties. The analysis of chemical composition and electronic properties were demonstrated by X-ray Photoelectron Spectroscopy (XPS, ThermoFisher, Waltham, MA, USA), with a b-monochromatic Alka source (*hv* = 1486.6 eV, 15 mA, 15 kV).

## 3. Results and discussion

### 3.1. Effect of Different Mass Fraction of Cellulose on Carrier Performance

**Figure 3:**
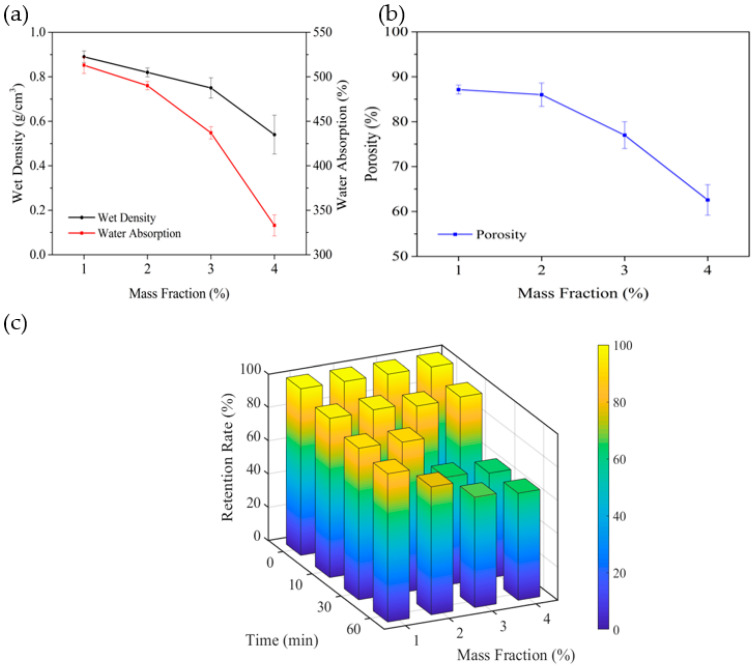
Effect of cellulose on water absorption: wet density (**a**), porosity (**b**), and retention (**c**) of the carrier.

The effects of mass fraction of cellulose on the carriers’ water absorption, wet density and porosity are shown in Figure 3a,b. The values of wet density, water absorption and porosity were 0.89 g/cm^3^, 513% and 87.14%, respectively, when mass fraction of cellulose was 1%. With the increasing of mass fraction of cellulose, all the values decreased gradually to 39.3%, 35.0% and 28.2%, respectively, compared with mass fraction of 4% to 1%. Cellulose is the basic framework of the carriers, and its fluffy internal structure plays an important role in forming sufficient pores in the carriers [24], which will effectively prevent the collapse of pores and being squeezed by the surrounding non-solidified solution. An appropriate amount of cellulose in carriers where mass fraction is 1% in this paper ensures more internal pores. Bagasse cellulose is hydrophilic [25], and a large amount of water can form hydrogen bonds with cellulose, effectively enhancing the ability to adsorb and store water. While the amount of cellulose increases gradually, especially mass fraction of 4%, the internal structure will become tighter and the wall of pores will become thicker. Thus, the pores inside the carriers take less remaining space correspondingly, leading to a decrease in the water absorption and porosity of carriers.

The change in retention rates of carriers with different mass fraction and test times is shown in Figure 3c. Within the test time of 10 min, the retention rate of carriers reaches 100%, whatever the type of different mass fraction of cellulose. The retention rate of carriers with mass fraction of 1% is 90.2% when the test time is up to 60 min, while that of others is less than 90.0%. Dissolving the efficiency of cellulose in ZnCl_2_ solution probably decreases gradually due to the increasing amount of cellulose. More undissolved cellulose leads to forming cellulose particles and agglomeration inside the carriers, breaking the stability of three-dimensional-net structure waved by cellulose and absorbent cotton and producing unbalanced force [26]. Therefore, the retention rate of carriers declines gradually with the increase in the mass fraction of cellulose. Instead of a high proportion, cellulose by the appropriate proportion of 1% interweaves with absorbent cotton to form a uniform three-dimensional mesh structure, with a strong ability to resist shear forces to achieve a higher retention rate of 90.2%. Therefore, the optimal mass fraction of cellulose is 1%.

### 3.2. Effect of Different Dosages of Absorbent Cotton on Carrier Performance

**Figure 4:**
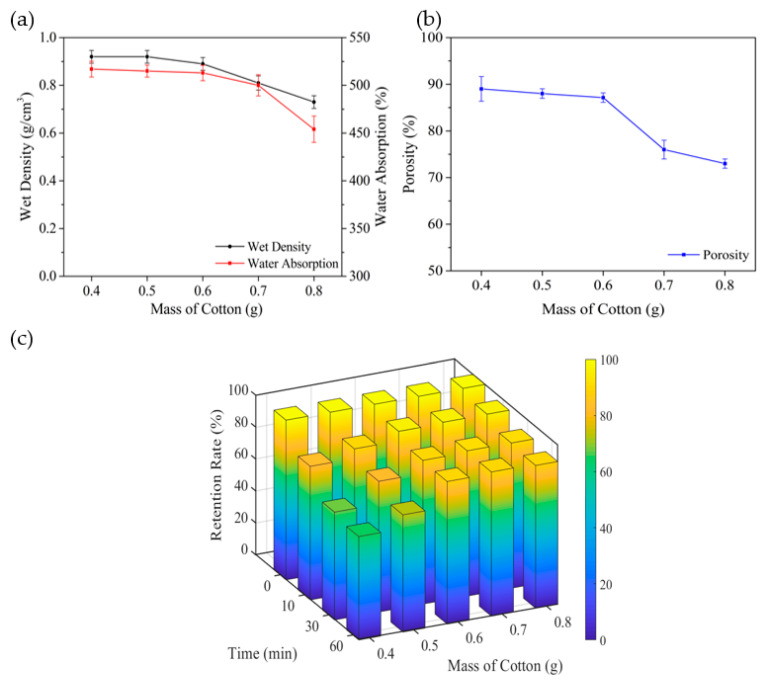
Effect of cotton on water absorption: wet density (**a**), porosity (**b**), and retention (**c**) of the carrier.

The effects of different dosages of absorbent cotton on the carriers’ performances are shown in Figure 4. With the amount of absorbent cotton from 0.4 g to 0.6 g, the values of the wet density, water absorption and porosity have a bit change and maintain the variation between 0.89 g/cm^3^ and 0.93 g/cm^3^, 512.0% and 520.0%, 87.0% and 90.0%, respectively. The amount of absorbent cotton has a further improvement to 0.8 g, while all of the values get a significant decline by 18.0%, 11.5% and 16.2% compared to the dosage of 0.6 g. This phenomenon is explained similarly to cellulose. The increasingly absorbent cotton cannot be sufficiently dissolved, contributing to cotton aggregation and destroying the three-dimensional mesh structure of the carriers [27], which leads to a decrease in porosity and water absorption.

Figure 4c shows the change in carrier retention rate at different dosages of absorbent cotton with test time from 0–60 min. The retention rates of carriers at dosages of 0.4 g and 0.5 g gradually decrease to 64.8% and 73.4% after testing for 60 min, while the retention rates remain more than 90.0% when the dosages vary from 0.6 g to 0.8 g. The higher the dosages of absorbent cotton are, the higher the strength of cellulose carrier is [27]. Although the strength of carriers is improved with a large dosage of cotton, the number of pores would decrease because the more compact structure of carriers and three-dimensional mesh structures will be destroyed by the undissolved cotton. Therefore, to ensure adequate porosity, the optimal dosage of absorbent cotton in this paper is 0.6 g.

### 3.3. Effect of Different Dosages of Na_2_SO_4_ on Carrier Performance

**Figure 5:**
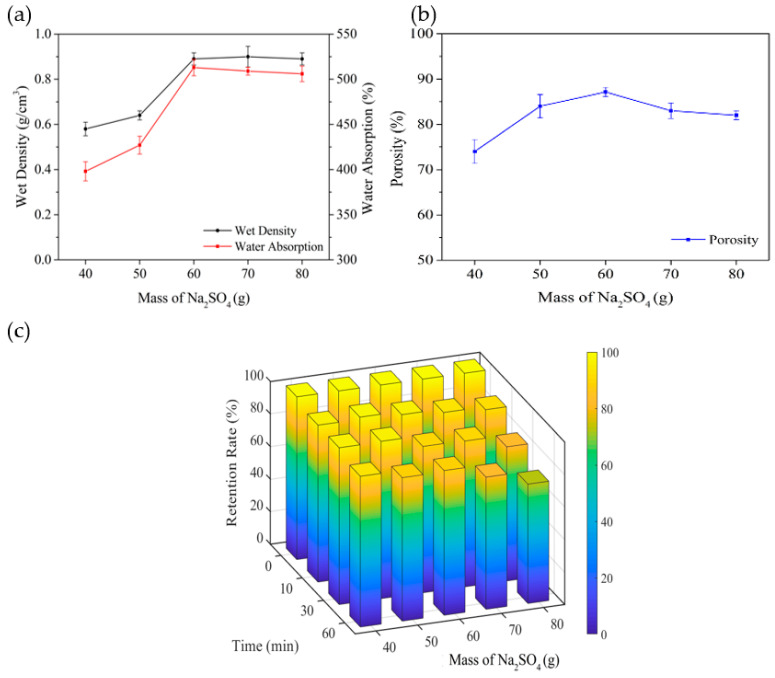
Effect of Na_2_SO_4_ on water absorption: wet density (**a**), porosity (**b**), and retention (**c**) of the carriers.

As shown in Figure 5, the values of wet density, water absorption and porosity of carriers, being exactly 0.89 g/cm^3^, 513% and 87.14%, respectively, reach a maximum when the dosage of Na_2_SO_4_ is 60 g. As a contributor of pores, Na_2_SO_4_ affects the number of pores in the carriers to a certain extent [28]. Theoretically, the more Na_2_SO_4_ is used, the fuller the porous structure and higher porosity in carriers will be, which is consistent with the evidence in Figure 5 when dosage varies from 40 g to 60 g. In addition, with the increase in Na_2_SO_4_ dosage, porosity and pore size, more hydrogen bonds are formed with water molecules and cellulose, to improve water absorption and wet density. This study is consistent with previous research results [26]. When the dosage is more than 60 g, the wet density and water absorption rate change little, and the porosity decreases slightly. This phenomenon comes from the porous collapse during freeze drying, when the Na_2_SO_4_ occupies more space in the carriers and the relatively thin supporting pore wall will collapse [27].

The retention rate of carriers still maintains a value more than 90.00% after testing for 60 min with the dosage of Na_2_SO_4_ increasing from 40 g to 60 g. With the addition of 70 g and 80 g, the retention rated decreases by 9.8% and 18.6% compared to that of 60 g. Carriers possess less pores and a thicker porous wall that makes the structure more compact, and a large number of hydrogen bonds forms into cellulose and cotton appearing with the dosage of more than 60 g, which gives the carriers a stronger ability against hydraulic shear forces. With a dosage of less than 60 g, the probability of collapse happening rises significantly on account of the porous wall becoming thinner, making the retention rate drop. Therefore, the dosage of 60 g is the optimal one for carriers.

### 3.4. Performance of TiO_2-_Coated Cellulose-Type Carrier

**Figure 6:**
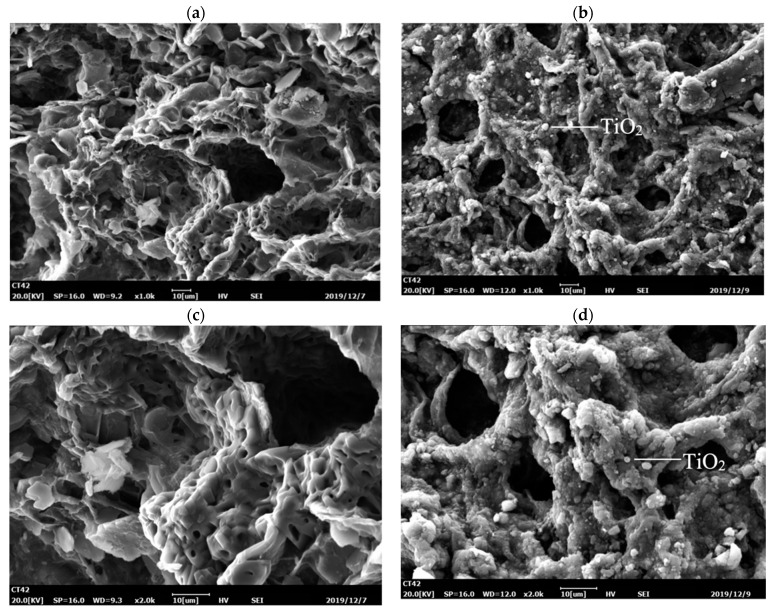
SEM images of carrier before and after loading nano-TiO_2: (_**a**,**c)** with magnification of 1.0 k and 2.0 k, respectively, and without nano-TiO_2_ loaded; (**b**,**d)** with magnification of 1.0 k and 2.0 k, respectively, and with nano-TiO_2_ loaded.

**Figure 7:**
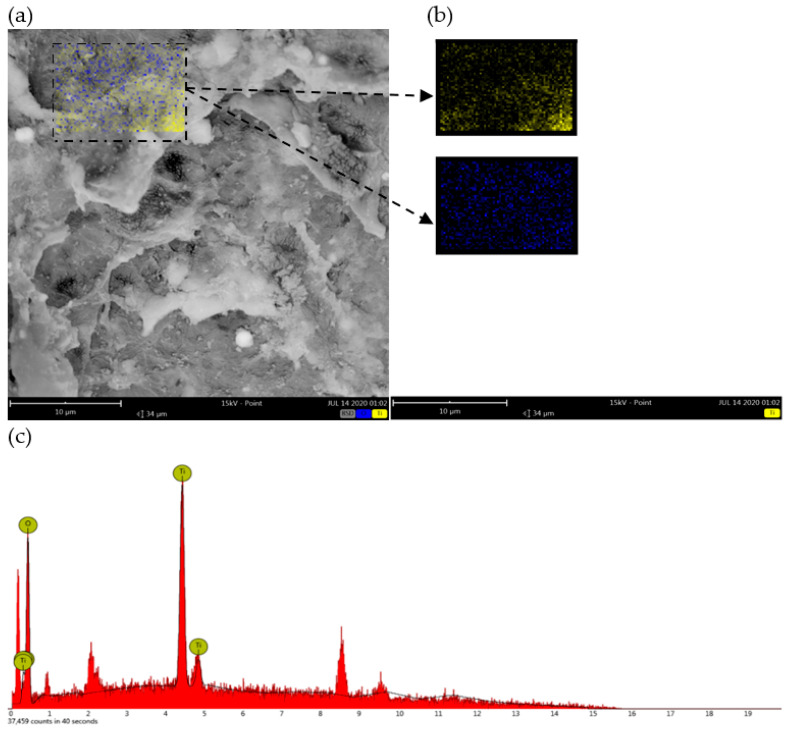
EDS image of nano-TiO_2_-cellulose carrier: (**a**) Image of EDS, (**b**) Element distribution, (**c**) Energy spectrum of elements).

The SEM images of carriers prepared for the optimal conditions are shown in Figure 6. Comparing the surface morphology of carriers before and after coating with nano-TiO_2_, it can be clearly seen that a large amount of nano-TiO_2_ has been coated into the carriers prepared by the method of a low-temperature process shown above, where the surface becomes rougher after coating nano-TiO_2_ than in the original carriers. Additionally, whether carriers are coated with nano-TiO_2_ or not, the pores of carriers are constructed with different diameters varying from 2 µm to 20 µm, indicating that the catalyst does not cover the pores of carriers and giving the possibility of growth and reproduction of microorganisms in the interior of the carriers. Moreover, EDS of nano-TiO_2-_coated cellulose-type carriers show that the main elements on the surface titanium and oxygen element in Figure 7 and the amount of the titanium element is approximately twice as much as the oxygen element, which proves that nano-TiO_2_ is successfully loaded onto the surface of carriers.

**Figure 8:**
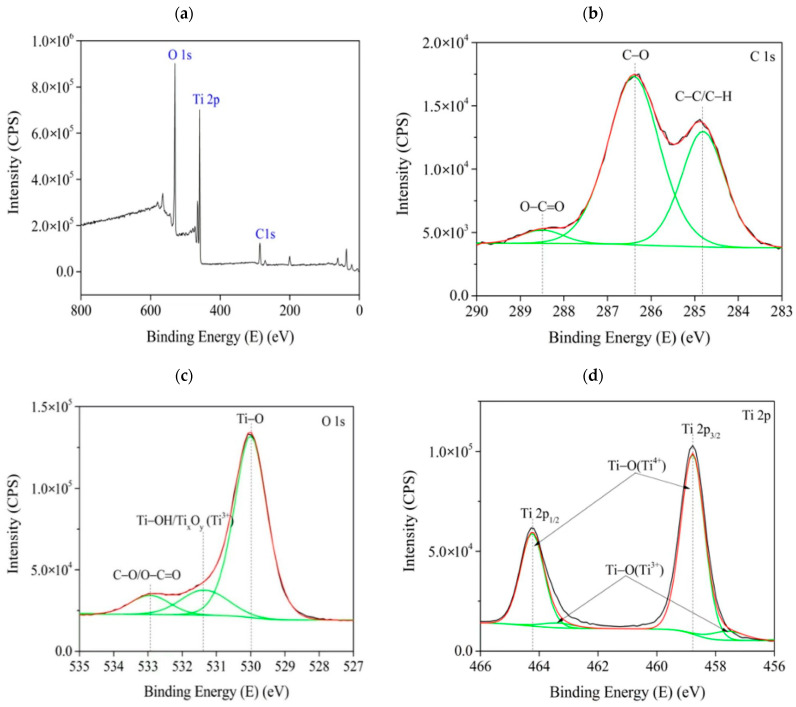
XPS image of TiO_2_-cellulose carrier: (**a**) survey spectra, (**b**), (**c**), and (**d**) are detailed spectra of C, O, and Ti, respectively).

To further confirm the presence of phase nano-TiO_2_ on the surface of the carriers, the chemical composition and electronic properties that were obtained from XPS analysis are shown in Figure 8. The survey spectra display the main signals from Ti, O and C (Figure 8a), and more specific properties acquired from a detailed spectrum (Figure 8b–d). Considering the composition of carriers, the C1s peak is mainly attributed to cellulose and cotton. The peaks at 532.09 eV and 532.97 eV, respectively, correspond to C–O and O–C=O bonds, associated to functional groups; for example, hydroxyl and carboxyl constructed in cellulose and cotton. The structure of the carbon skeleton is demonstrated by the bond between C–C/C–H with the energy of 284.78 eV. Except for the substrate grown catalyst, a little of the signals of adjacent to the C1s peak maybe comes from carbon contamination as the sample exposing to air [29].

Analyzing the detailed spectrum of O1s core line, it was found that the peak could be deconvoluted into three components located at 532.98 eV, 531.38 eV and 529.98 eV, respectively, which originate from the titanium oxide and oxygen-containing functional groups of carriers and the surface of catalyst. The first component, consistent with the one of C1s peak, corresponds to C–O/O–C=O bonded with functional groups of cellulose molecule. The second emergence means that a low-valence Ti oxidized has been generated in catalyst, such as Ti–OH bond (Ti hydroxide species) and Ti_x_O_y_. The last component originates from the bond between Ti–O combining O_2_^-^ and Ti^4+^ in nano-TiO_2_.

The detailed spectrum of Ti 2p core line has been deconvoluted into four components, including two prominent peaks of Ti 2p3/2 and Ti 2p1/2 positioned at 458.78 eV and 464.48 eV, respectively, corresponding to Ti^4+^ in titanium dioxide [30,31]. Moreover, two weaker peaks locate closely on the shoulders of prominent peaks at 457.58 eV and 463.48 eV due to the presence of oxygen vacancy and a low-valence Ti oxidized as described as lattice defects, which can improve the efficiency of photocatalysis and widen the range of excitation wavelength to the visible from the ultraviolet [32], giving a feasible explanation of visible-light reaction to the catalyst used. Therefore, the catalyst successfully loaded onto the surface of carriers.

### 3.5. Analysis of Photocatalytic Properties of Cellulose Composite Carriers

**Figure 9:**
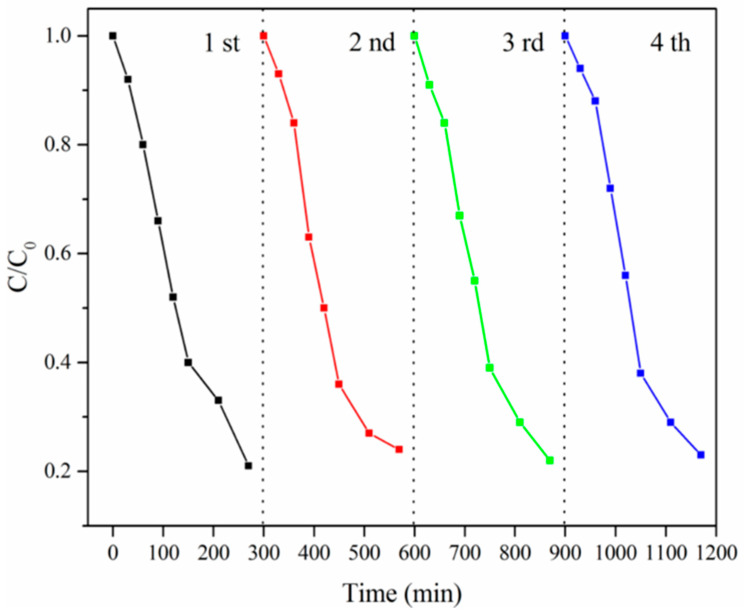
Cycle experiment of RhB degradation.

The above studies confirmed that nano-TiO_2_ was loaded on the surface of the cellulose carriers, and the degradation experiment of methylene blue showed that titanium dioxide had good photocatalytic activity [33]. In addition, the band gap energy of TiO_2_ is 3.15 eV, and this low band gap energy makes TiO_2_ have a wide range of UV Vis spectra, mainly in the range of 350–600 nm; it further shows the photocatalytic activity of TiO_2_. In order to further determine the photocatalytic performance of the nano-TiO_2_–cellulose composite carrier and the reuse stability of the composite carrier, 15 mg/L RhB solution was used as the target pollutant for 4 cycles in this study. The experimental results are shown in Figure 9, where it can be seen from the figure that the degradation rate of RhB by the nano-TiO_2_–cellulose composite carrier can reach 80.00% within 5 h. Four rounds of RhB repeated degradation experiments were carried out, and the degradation rate of RhB was basically stable at 80.00%, which proved that the nano-TiO_2_–cellulose composite support had good photocatalytic performance and stability under the conditions of this study. In Table 1, compared with other materials, it is clear that the cellulose carrier loaded with TiO_2_ has good photocatalytic activity, shorter time-consumption and higher degradation efficiency.

### 3.6. Degradation of 1,2,4-TCB in ICPB

**Figure 10:**
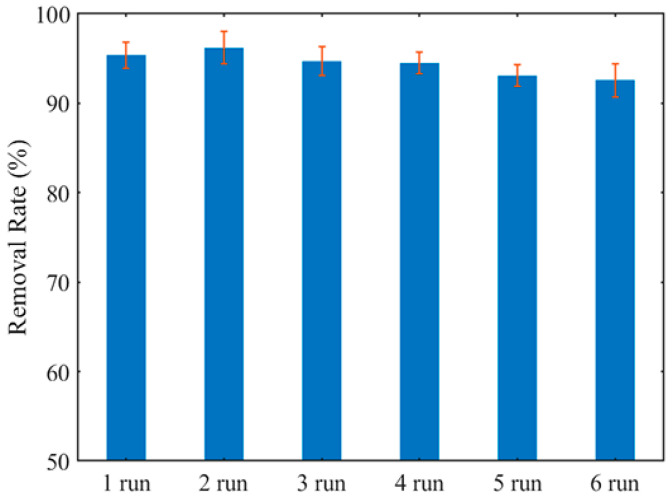
Change curve of 1,2,4-TrCB concentration in six consecutive batches of ICPB system.

**Figure 11:**
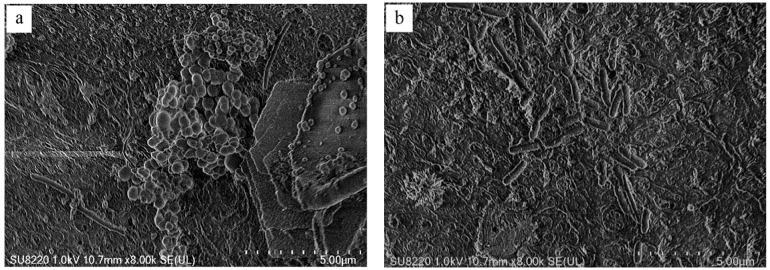
SEM images of biofilms located in the core of the carrier before (**a**) and after (**b**) ICPB reaction.

The system of ICPB was constructed by the carrier coated catalyst and loading loaded biofilm, and 1,2,4-TCB was selected as an object for testing performance of the carriers in ICPB. The six-cycling experiments for the same batch of carriers are shown in Figure 10 with an operation time of 7 h. The degradation rate of 1,2,4-TCB in ICPB at first gets up to 95.4% and stabilizes above the level of 92.0% generally. With the carrier cycle experiment, the degradation rate gradually decreased, and the sixth decreased by 2.8% compared with the first cycle experiment; this indicates that the cellulose-type carriers can be applied to construct the system of ICPB. In addition, a slight decline in degradation rates of 1,2,4-TCB after cycles can come from falling off of a catalyst struck constantly by the stirrer, and the high degradation rate in the 2nd cycle may be related to the aggregation and accumulation of TiO_2_ on the surface of a cellulose carrier. In order to confirm whether the microorganism attached to the carriers can still load on that after six-cycles running, the images of carriers were taken by SEM and are shown in Figure 11. Before the carriers participate in the degradation, a large amount of microorganism attached to the carriers (Figure 11a,b) and after the six-cycles, abundant microorganisms were still there in the carriers, which illustrates that the carriers can shelter microorganisms from the damage to active species and radiation of a light source, consistent with the research of Xiong [35]. Therefore, the cellulose-type carrier has been successfully used to construct the system of ICPB and achieve effective degradation of 1,2,4-TCB.

## 4. Conclusions

In this paper, sugarcane cellulose, absorbent cotton and anhydrous sodium sulfate were used as materials to prepare a cellulose porous carrier. The performance of porous the carriers were investigated by taking water absorption, wet density, porosity and retention as indicators, and the preparation process was optimized; nano-TiO_2_ was loaded on it. The results showed that the best preparation conditions were cellulose mass fraction of 1.0%, absorbent cotton of 0.6 g, Na_2_SO_4_ of 60 g. The SEM, EDS and XPS characterization showed that nano-TiO_2_ could be effectively loaded onto the surface of a cellulose carrier, and the surface and pore structure of the carriers provided conditions for microbial attachment. The degradation rate of RhB in four cycles was more than 80%, which indicates that a nano-TiO_2_ cellulose carrier has good photocatalytic performance, which lays a good foundation for the subsequent ICPB system to achieve efficient degradation of 1,2,4-TCB.

## Figures and Tables

**Table 1:** The comparison of photocatalytic activity of different materials.

Carrier	Catalyst	Pollutant	Pollutant Concentration	Light Source	Time	Efficiency
/	CdS/TiO_2_ [19]	1,2,4-TCB	0.1 mol/L	UV	7.5 h	32.60%
Ceramic porous carrier	TiO_2_ [15]	2,4-DNT	50 mg/L	UV	60 h	78%
Sponge carrier	TiO_2_ [16]	2,4,5- TCP	50 μM	UV	6 h	94.2%~98.2%
Sponge carrier	Ag/TiO_2_ [34]	TCH	20 mg/L	visible	8 h	94%
Cellulose carrier	TiO_2_ [12]	MB	15 mg/L	UV	6 h	92.08%

## Data Availability

Not applicable.
